# Ovarian and Adrenal Androgens and Their Link to High Human Chorionic Gonadotropin Levels: A Prospective Controlled Study

**DOI:** 10.1155/2014/191247

**Published:** 2014-11-23

**Authors:** René Rodríguez-Gutiérrez, Jesús Zacarías Villarreal-Pérez, Felipe Arturo Morales-Martinez, René Rodríguez-Guajardo, Gloria González-Saldivar, Leonardo G. Mancillas-Adame, Neri Alejandro Alvarez-Villalobos, Fernando Javier Lavalle-Gonzalez, José Gerardo González-González

**Affiliations:** ^1^Endocrinology Division, University Hospital “Dr. José E. González”, School of Medicine, Autonomous University of Nuevo León, Francisco I. Madero and Gonzalitos s/n, 64460 Monterrey, NL, Mexico; ^2^Obstetrics and Gynecology Department, University Hospital “Dr. José E. González”, School of Medicine, Autonomous University of Nuevo León, Francisco I. Madero and Gonzalitos s/n, 64460 Monterrey, NL, Mexico; ^3^Clinical Research Unit, University Hospital “Dr. José E. González”, School of Medicine, Autonomous University of Nuevo León, Francisco I. Madero and Gonzalitos s/n, 64460 Monterrey, NL, Mexico

## Abstract

*Background*. Although the association between human chorionic gonadotropin (hCG) and hyperandrogenism was identified more than 40 years ago, relevant questions remain unanswered. *Design and Methods*. We conducted a prospective, longitudinal, and controlled study in 23 women with a diagnosis of a complete hydatidiform mole (HM). *Results*. All participants completed the study. Before HM evacuation mean hCG was markedly higher in the cases than in the control group (*P* ≤ 0.001). Free testosterone (T) and dehydroepiandrosterone sulfate (DHEA-S) were found to be higher in the cases (2.78 ± 1.24 pg/mL and 231.50 ± 127.20 *μ*/dL) when compared to the control group (1.50 ± 0.75 pg/mL and 133.59 ± 60.69 *μ*/dL) (*P* = 0.0001 and 0.001), respectively. There was a strong correlation between hCG and free T/total T/DHEA-S concentrations (*r* = 0.78; *P* ≤ 0.001, *r* = 0.74;  *P* ≤ 0.001, and *r* = 0.71;  *P* ≤ 0.001), respectively. In the cases group 48 hours after HM evacuation, hCG levels were found to be significantly lower when compared to initial levels (*P* = 0.001) and free T and DHEA-S declined significantly (*P* = 0.0002 and 0.009). *Conclusion*. Before uterus evacuation, hCG, free T, and DHEA-S levels were significantly higher when compared with controls finding a strong correlation between hCG and free T/DHEA-S levels. Forty-eight hours after HM treatment hCG levels declined and the difference was lost. A novel finding of our study is that in cases, besides free T, DHEA-S was also found to be significantly higher and both the ovaries and adrenal glands appear to be the sites of this androgen overproduction.

## 1. Introduction

Gestational trophoblastic disease (GTD) includes a heterogeneous group of pathologies that arise from anomalous proliferation of trophoblastic cells [1, 2]. Ninety percent of cases are secondary to hydatidiform mole (HM), which is classically a benign, noninvasive tumor with an excess of paternal chromosomes [3]. All forms of GTD are characterized by secretion of human chorionic gonadotropin (hCG), which is a glycoprotein conformed by alpha and beta subunits. The alpha subunit is identical to other glycoproteins such as thyroid stimulating hormone (TSH), follicle-stimulating hormone (FSH), and luteinizing hormone (LH). On the other hand, the beta subunit is unique to hCG and confers its biological specificity. However, there is still considerable homology, in the beta subunit, between these glycoproteins [4, 5]. Moreover, their corresponding receptors are all members of the G-protein couple receptor family and contain a similar aminoterminal ectodomain. It has been recognized that due to these biochemical and molecular similarities, supraphysiological concentrations of one of these hormones can generate the biological effect of the other. This relatively high sequence identity between the hormone-binding domains has been called “spillover phenomenon” and has been classically described during pregnancy as being responsible for hCG-mediated hyperthyroidism [6–8].

Although the association between hCG and hyperandrogenism was identified more than 40 years ago, relevant questions remain unanswered. In fact, there is conflicting data of whether this association is consistent [9–11]. Additionally, if hyperandrogenism disappears, and if it does, the potential correlation between hCG and androgen levels, the clinical impact that this association might have, and whether other androgens besides total testosterone (T) are also elevated are questions that remain unanswered. Furthermore, if the underlying mechanism of this enhanced androgen production is secondary to a high hCG concentration (spillover phenomenon), direct trophoblastic production, decreased aromatase activity in the placenta, and/or production of an androgen binding protein by the HM are still not well elucidated [12–16].

Accordingly, a prospective, longitudinal, and controlled study was carried out to evaluate serum ovarian and adrenal androgens in patients with a diagnosis of complete HM before and after uterus evacuation. The primary endpoint was to define the behavior of ovarian and adrenal androgens during and after high hCG concentrations. Secondary objectives were to compare serum androgen levels with gestational age-matched controls and to identify if there was a correlation between hCG and androgen levels. Additionally, in cases with high androgen levels, we sought to determine if this was reversible, when it happened, and if there was any correlation between biochemical and clinical hyperandrogenism.

## 2. Subjects and Methods

### 2.1. Subjects

After obtaining approval from the Research and Ethics Committee of the “Dr. José E. González” University Hospital, we began the study. Signed informed consent was obtained from each participant before entering the study. Patients were recruited through the Obstetrics and Gynecology Clinic of our institution. A total of 23 women with an age ≥18 years and a diagnosis of a complete HM were included. A case was defined by characteristic clinical features (vaginal bleeding, enlarged uterus, and pelvic pain), higher hCG levels than those observed in normal or ectopic pregnancies of the same gestational age, and distinctive ultrasound findings, such as absence of an embryo or fetus, theca lutein cysts, no amniotic fluid, and a central heterogeneous mass with a vesicular pattern (snowstorm pattern) [17]. Diagnosis was confirmed, in all cases, by histologic examination of tissue [18]. We excluded women with a previous diagnosis of polycystic ovary syndrome, 21-hydroxylase-deficient nonclassic adrenal hyperplasia, obesity, impaired glucose tolerance, any other disorder associated with hyperandrogenism, and if they had used any medication in the last 24 months that might interfere with androgen metabolism, transport, or the hypothalamic-pituitary-gonadal axis. An age, weight, and gestational age-matched control group (*n* = 23) of healthy pregnant women was recruited from the Obstetrics and Gynecology Clinic.

### 2.2. Study Protocol

After the diagnosis of a molar pregnancy and before HM evacuation, complete clinical history, physical examination, and measurement of anthropometric indexes, such as weight, height, body mass index (BMI), and waist and hip circumference, were performed in all participants. Clinical hyperandrogenism was defined as the presence of androgenic alopecia, hirsutism, or acne. One experienced independent blinded observer assessed the presence of hirsutism, acne, male pattern baldness, and/or seborrhea. Subsequently, an intravenous plastic catheter was inserted into a forearm vein and baseline blood samples were drawn for the measurement of FSH, LH, hCG, total T, free T, androstenedione, dehydroepiandrosterone sulfate (DHEA-S), TSH, total thyroxin (T4), free thyroxin (FT4), and liothyronine (T3). Following the same protocol, this same hormone panel was repeated, in all cases, 48 hours and 2 weeks after uterus evacuation. In the control group, hormones were measured only once (gestational age matched). All samples were immediately centrifuged, separated, and frozen at −20°C until used for the hormonal assays.

### 2.3. Measurements

Human chorionic gonadotropin was measured using an immunochemiluminometric assay (ICMA) specific for the beta hCG subunit with an analytical sensitivity of 2 mIU/L (Cobas 6000 analyzer series, Roche Diagnostics Limited, Risch-Rotkreuz, Switzerland). FSH and LH were determined by immunoassay (Cobas e411 analyzer, Roche Diagnostics, Mannheim, Germany). Total T and free T were measured using liquid chromatography/tandem mass spectrometry (Quest Diagnostics Nichols, Chantilly, VA). Androstenedione and DHEA-S were determined by enzyme-linked immunoabsorbent assay (BioVendor Research and Diagnostic Products, Asheville, NC). Intra-assay variation coefficients were 5.2 and 9.3%, respectively. Thyroid function testing included measurement of T4, FT4, T3, and TSH. Thyroxin, FT4, and T3 were determined using a commercial kit for electrochemiluminescence (Modular Analytics E170, Roche/Hitachi Diagnostics, Mannheim, Germany). Intra-assay variation coefficients were 3.7, 3.4, 4.2, and 2.7%, respectively. TSHwas measured using a commercial electrochemiluminescence kit (Modular Analytics E170, Roche/Hitachi Diagnostics) with an intra-assay variation coefficient of 3.2%. Cortisol was measured with a radioimmunoassay kit (Elecsys 2010, Roche/Hitachi Diagnostics) with an intra-assay variation coefficient of 1.6%. All samples were assessed twice. The Ferriman-Gallwey modified scale was used for hirsutism assessment [19]. Patients with scores of 8 or greater were considered hirsute. Acne was graded by a scoring system from 0 to 3 and alopecia was evaluated by the Ludwig scoring system [20, 21]. Anthropometric measurements were performed according to the International Society for the Advancement of Kinanthropometry (ISAK) and National Institutes of Health suggestions [22]. Weight and height were determined on a Seca 700 calibrated mechanical scale with a stadiometer (TAQ, Sistemas Médicos, Mexico City, Mexico) and BMI was calculated with the following formula: BMI = (weight in kg)/(height in m)^2^.

### 2.4. Statistical Analysis

All results are reported as mean ± standard deviation unless otherwise stated. A *P* value equal or less than 0.05 was considered statistically significant. Descriptive statistical analysis was used for quantitative variables and measures of central tendency and dispersion. In the case of qualitative variables, frequencies were obtained. To compare differences between basal and follow-up hormone profiles within each patient group and between patients and controls paired and unpaired, Student's *t*-tests were used, respectively. Correlation analyses were done using Pearson's correlation coefficient. Values from 0.99 to 0.90 defined a very strong, 0.89 to 0.70 a strong, 0.69 to 0.40 a moderate, and 0.3 to 0.1 a weak correlation [23]. Statistical analysis was performed with IBM SPSS Statistics 20.0 software (SPSS, Inc., Armonk, NY).

## 3. Results

### 3.1. Study Population

The demographic characteristics of the participants are shown in Table 1. A total of 46 pregnant women, twenty-three in the case and in the control groups were included in the study. All participants completed the study. Mean age was 26.1 ± 4.8 years in the cases and 26.5 ± 5.6 in the controls with a gestational age of 13.1 ± 1.51 weeks and 12.0 ± 1.9, respectively, (*P* = 0.75 and 0.54). Mean BMI was within normal range in both groups and there were no significant differences between any of the anthropometric measures.

### 3.2. Assessment before HM Evacuation: Cases versus Controls

Before HM evacuation mean hCG was markedly higher in the cases (126, 897 ± 39, 761 mIU/mL) than in the control group (52, 148 ± 26, 003 mIU/mL) (*P* = 0.0001). Free T and DHEA-S were found to be statistically higher in the cases (2.78 ± 1.24 pg/mL and 231.50 ± 127.20 *μ*/dL) when compared to the control group (1.50 ± 0.75 pg/mL and 133.59 ± 60.69 *μ*/dL) (*P* = 0.0001 and 0.001, resp.). There was a tendency for total T to be higher in the cases (126.19 ± 56.96 ng/dL) when compared with the controls (104.02 ± 33.27 ng/dL), but this was not significant (*P* = 0.10). FT4 and T3 were significantly higher as well and TSH was lower in the cases when compared with controls. There were no differences in FSH, LH, androstenedione, cortisol, and T4 serum levels between groups. See Table 2.

### 3.3. Hormonal Assessment before and after HM Evacuation

#### 3.3.1. Before versus 48 Hours after HM Evacuation

In the cases group, 48 hours after HM evacuation, hCG concentration was found to be significantly lower (126, 897 ± 39, 761 versus 26, 636 ± 18, 155 mIU/mL) when compared to its initial concentration (*P* = 0.001). Free T and DHEA-S declined significantly (2.78 ± 1.24 versus 1.54 ± 0.71 pg/mL and 231.50 ± 127.20 versus 161.43 ± 71.68 *μ*/dL, resp.) (*P* = 0.0002 and 0.009). Cortisol was found to be higher (181.70 ± 111.08 versus 121.75 ± 56.4 nmol/L) (*P* = 0.04) and T3 declined significantly (228.88 ± 93.87 versus 135.90 ± 38.69 ng/dL) (*P* = 0.001) after the first 48 hours. See Table 3.

#### 3.3.2. Before versus 2 Weeks after HM Evacuation

As expected, two weeks after HM evacuation FSH, LH and TSH were found to be higher. Total T, free T, androstenedione, DHEA-S, hCG, T4, FT4, and T3 were all found to decline significantly after 2 weeks of HM evacuation. See Table 3.

### 3.4. Assessment after 2 Weeks of HM Evacuation: Cases versus Controls

Two weeks after HM evacuation total T was found to be significantly higher in the control group (52.50 ± 32.90 versus 104.02 ± 33.27 ng/dL) (*P* = 0.001). Free T was within normal range in both groups without any statistical difference (1.25 ± 0.77 versus 1.50 ± 0.75) (*P* = 0.25). Androstenedione and DHEA-S were not different from each other as well. T4 and T3 were significantly higher in pregnant women. See Table 4.

### 3.5. Correlation between hCG and Androgens

**Figure 1:**
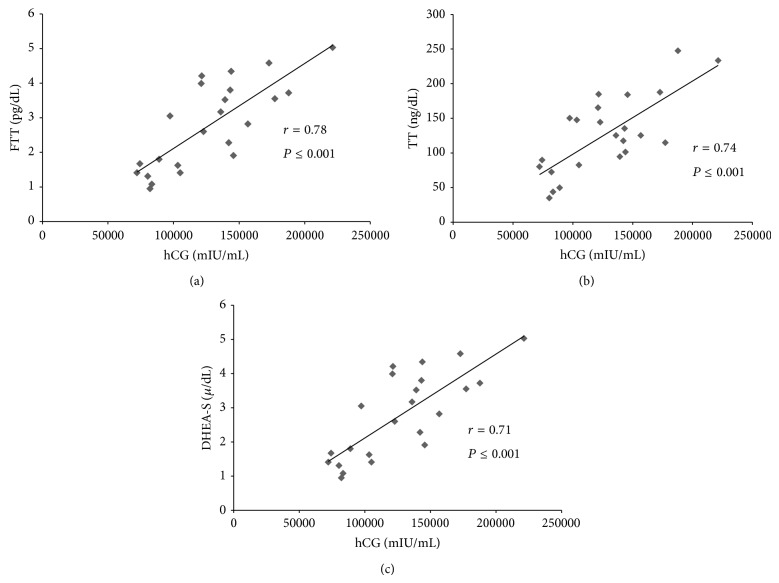
Coefficient of correlation between (a) hCG and free testosterone (FT), (b) hCG and total testosterone (TT), and (c) hCG and dehydroepiandrosterone sulfate (DHEA-S).

There was a positive, strong, and significant correlation between hCG and free T concentrations (*r* = 0.78; *P* ≤ 0.001). When this same analysis was made between hCG and total T/DHEA-S, a strong and significant correlation was also found (*r* = 0.74, *P* ≤ 0.001; *r* = 0.71, *P* ≤ 0.001, resp.). See Figure 1.

### 3.6. Clinical Signs of Hyperandrogenism

When compared before HM evacuation, modified Ferriman-Gallwey, acne, and androgenic alopecia scores were not different between the case and control groups. In the longitudinal comparison made within the cases group there were also no differences in any of the previous signs of androgen excess.

## 4. Discussion

In this prospective, observational, and controlled study we found that in women with complete HM, before uterus evacuation, hCG, free T, and DHEA-S were significantly higher when compared with age-, weight-, and gestational age-matched controls. A strong correlation between hCG and total T/free T/DHEA-S concentrations was found and forty-eight hours after HM treatment hCG levels decreased and both free T and DHEA-S concentrations returned to normal values. In the cases group, even though baseline total T and androstenedione had a tendency to be higher when compared to the control group, this was not significant. Two weeks after HM evacuation, all hormones including thyroid function tests returned to normal values and patients remained asymptomatic. Clinical signs of hyperandrogenism were not found to be present at any point of the study in either cases or controls.

The relationship between high hCG levels and ovarian and adrenal androgens in HM is still controversial. In 1972, Samaan et al. described high serum total T concentrations in 11 patients with trophoblastic disease. Total T was found to be particularly higher in the subgroup of patients with enlarged ovaries than in those with normal size. Both total T and hCG decreased after chemotherapy and/or oophorectomy. Nevertheless, total T was measured using the protein binding method. The authors did not exclude pathologies associated with androgen excess (polycystic ovary syndrome) and in the normal size ovary subgroup, 50% of the cases had total T levels that were below the mean of what they considered to be normal. No correlation analysis was made between hCG and androgens; neither free T or other androgens were measured nor were they compared to a control group [9]. Later, Dawood and Saxena measured total T and dihydrotestosterone in 14 women with HM and compared them with 16 gestational age-, but not age- and weight-matched controls. Total T levels ranged from 27 to 530 ng/dL, and in 42% of their cases (6/14) they were found to be within normal pregnancy levels. Dihydrotestosterone, as expected, was found to be higher in cases and they found no correlation between hCG and total T or dihydrotestosterone levels. There was no follow-up; DHEA-S, free T, and androstenedione were not measured, and data concerning possible clinical signs of hyperandrogenism, even though mentioned to be absent, were not clearly reported [10]. Later on, Chew et al. reported total T levels to be higher in HM patients when compared to normal pregnancies of corresponding gestation levels and to significantly fall, without specifying a precise time, after uterus evacuation. The trophoblast was proposed as the main source of androgen production secondary to the conversion of DHEA to total T within the molar trophoblast. No correlation was found between total T and uterus size and only a moderate/weak association was found between hCG and total T. Other androgens were not measured and clinical implications were not reported [11, 13, 24]. In our study, knowing that androgen levels in adult females change with age and corporal composition, women with a diagnosis of a complete HM were compared not only with gestational age- but also with age- and weight-matched controls. Free T and DHEA-S, in the presence of a high hCG concentration, were found to be significantly higher in cases than in controls; two days after uterus evacuation hCG immediately decreased and this difference was lost. Serum total T and androstenedione levels even though elevated, compared with normal serum levels, were not significantly different from the control group. Moreover, a strong and significant positive correlation was found between hCG and total T/free T/DHEA-S. Additionally, clinical manifestations of hyperandrogenism (hirsutism, androgenic alopecia, and acne) were meticulously examined and neither before nor after HM treatment were found to be different.

The source and mechanism by which androgen levels are elevated in HM is still not well elucidated [12, 14–16, 24]. Androgen production in women may be from an ovarian, adrenal, or peripheral conversion source. Total T is synthetized in the adrenal gland (25%), ovaries (25%), and 50% from androstenedione and DHEA-S conversion. Both glands equally produce androstenedione, but DHEA-S is produced solely in the adrenal gland and DHEA is produced 50% in the adrenal gland, 30% from conversion of DHEA-S, and 20% in the ovaries. Dihydrotestosterone is classically an intracellular androgen [25, 26]. During normal gestation total T progressively rises primarily due to an estrogen-induced increase in serum sex hormone-binding globulin (SHBG) and secondarily to a decreased clearance rate. Free T usually remains stable, DHEA-S concentrations decline due to an increase in its clearance rate, and androstenedione levels slightly increase [27–29].

In GTD, serum androgen-binding globulin (produced by the trophoblast) and SHBG have been proposed as a responsible mechanism for high total T levels [14–16]. This could explain high total T levels but not a high free T or DHEA-S concentration. In fact, in the latter, an inverse relationship between SHBG and DHEA-S has been described [30]. As well, it has been suggested that high androgen levels in HM could be explained by a conversion of DHEA to total T secondary to the presence of 17-hydroxysteroid dehydrogenase and 3-beta hydroxysteroid dehydrogenase in the trophoblast based on the fact that total T was found to be not different in patients with and without theca-lutein cysts. However, it is well known that the reported prevalence of theca-lutein cysts in complete HM is only 20–50% and that their absence does not preclude the lack of other theca interstitials cells within the ovaries. Additionally, in this report, adrenal androgen production was not explored [24]. A low aromatase activity with a consequent increase in total T levels was also described in an* in vitro *study in which the kinetic parameters of aromatase in microsomes from HM were compared to those of a normal early placenta. Enzymes from both sources were kinetically similar, but HM cells demonstrated lower enzyme efficiency (maximum velocity/*K*_*m*_ ratio). Unfortunately, no further studies were made in an* in vivo *model [12]. Finally, supraphysiological hCG levels have been associated with increased androgen production as hCG shares structural similarity with LH that under supraphysiological concentrations stimulate, via spillover phenomenon, LH receptors from the theca-interstitial cells [13, 31]. The fact that in our study total T, unlike free T, was found to be high but not different from controls makes us believe that high free T levels cannot be explained solely by an increase in serum SHGB, trophoblast produced androgen binding protein, or decreased aromatase activity, but rather by an increase in its production. Furthermore, due to the strong correlation that we found between hCG and free T, we can conclude that this could be explained, at least partially, by the stimulation of theca interstitials cells due to high hCG concentration (spillover phenomenon). Consistent with this, after uterus evacuation and parallel with hCG levels, free T rapidly deceased, and this difference was lost.

Not previously documented, a novel finding of our study was the DHEA-S elevation found in cases, which was significantly higher when compared to controls. The mechanism by which adrenal androgens, including DHEA-S, are regulated is not completely understood and even though ACTH is known to stimulate DHEA-S secretion, there are clinical scenarios such as a Cushing disease and ectopic ACTH syndrome that have reported DHEA-S concentrations to be normal or even low [32–34]. These inconsistencies indicate that factors, other than ACTH, play a role in adrenal androgen production regulation. Accordingly, it has been reported that adrenal glands express the LH receptor gene and that LH stimulates adrenal androgen secretion [32, 35–37]. Moreover, an hCG/LH receptor has been described in the entire zona reticularis and deeper layer of zona fasciculate of the adrenal cortex [38]. Consequently, the fact that in our study, hCG, DHEA-S, and free T were found to be elevated and knowing that 25% of total T and 100% of DHEA-S are produced in the adrenal glands and that free T elevation can be explained only by an increase in its production make us believe that the adrenal glands were, in addition to the ovaries, responsible for DHEA-S and free T elevation probably due to the stimulation of LH/hCG and LH receptors via spillover phenomenon. Supporting this mechanism, we found a strong and significant correlation between hCG and DHEA-S concentrations and cases were found to have a low TSH and high FT4/TT3 that are well known to be secondary to TSH receptor stimulation due to supraphysiologic hCG concentrations (spillover phenomenon) [6–8].

The clinical impact that high androgen levels might have in this scenario has been often overlooked. In our study, a profound physical examination was made before and after HM evacuation. Modified Ferriman-Gallwey, acne, and androgenic alopecia scores were not different in any point of the study between the case and control groups. This could be explained by various reasons. First, progesterone production increases considerably during pregnancy and due to its weak affinity for androgen receptors high levels might inhibit androgen binding to its receptor. Moreover, it has also a weak affinity for 5-alpha reductase that might have inhibited the conversion of total T into its active metabolite, dihydrotestosterone [39, 40]. Second, it is well known that the placenta has a massive capability to convert androgens to estrogens via aromatization [41]. Finally, it is possible that the time of exposure was not enough, as it is well known that due to their nuclear mechanism of action biochemical hyperandrogenism may take weeks and even months to express clinically [42].

## 5. Conclusion

In conclusion, before uterus evacuation, in women with a diagnosis of complete HM, hCG, free T, and DHEA-S concentrations were significantly higher when compared with controls finding a strong and significant positive correlation between hCG and free T/DHEA-S concentrations. Forty-eight hours after HM treatment hCG levels declined and the difference was lost. High serum levels of free T and DHEA-S cannot be explained solely by an increase in SHGB, trophoblast produced androgen binding protein, or decreased aromatase activity, but rather by an increase in their production. A novel finding of our study is, that in cases, besides free T, DHEA-S was also found to be significantly higher and both ovaries and adrenal glands appear to be the sites of this androgen overproduction. This could probably be explained by stimulation of LH and hCG/LH receptors by supraphysiologic hCG concentrations (spillover phenomenon).

## Figures and Tables

**Table 1:** Demographic characteristics.

	Cases	Controls	*P* value
Number of participants	23	23	—
Age (yr.)	26.1 ± 4.8	26.5 ± 5.6	0.75
Gestational age (w.)	13.1 ± 1.51	12.0 ± 1.9	0.54
Weight (Kg.)	61.4 ± 13.77	61.72 ± 9.8	0.95
Height (m.)	1.5 ± 0.03	1.58 ± 0.06	0.97
BMI	24.59 ± 5.6	24.5 ± 4.1	0.93

yr.: years; w.: weeks; Kg.: kiligrams; m.: meters; BMI: body mass index.

Plus-minus values are mean ± standard deviation.

^*^Significant *P* ≤ 0.05.

**Table 2:** Cases versus controls before HM evacuation.

	Cases (*n* = 23)	Controls (*n* = 23)	*P* value
FSH (UI/L)	0.1 ± 0.02	0.1 ± 0.03	0.83
LH (IU/L)	0.1 ± 0.03	0.1 ± 0.02	0.76
hCG (mIU/mL)	126,897 ± 39,761	52,148 ± 26,003	<0.001^*^
TST (ng/dL)	126.19 ± 57.35	04.02 ± 33.27	0.10
FTST (pg/mL)	2.78 ± 1.24	1.50 ± 0.75	<0.001^*^
AN (ng/mL)	3.88 ± 2.22	3.55 ± 1.42	0.55
DHEA-S (*μ*/dL)	231.48 ± 124.59	133.59 ± 60.69	0.001^*^
Cortisol	181.70 ± 111.08	188.53 ± 97.72	0.05
TSH (mU/L)	0.81 ± 0.69	2.69 ± 3.71	0.03^*^
T4 (*μ*g/dL)	13.98 ± 4.97	11.84 ± 1.91	0.06
FT4 (ng/dL)	1.73 ± 1.12	1.07 ± 0.14	0.007^*^
T3 (ng/dL)	228.88 ± 93.87	181.60 ± 34.04	0.02^*^

HM: hydatidiform mole; FSH: follicle-stimulating hormone; LH: luteinizing hormone; hCG: human chorionic gonadotropin; TTST: total testosterone; FTST: free testosterone; AN: androstenedione; DHEA-S: dehydroepiandrosterone sulfate; TSH: thyroid stimulating hormone; T4: thyroxin; FT4: free thyroxin; T3: liothyronine.

Plus-minus values are mean ± standard deviation.

^*^Significant P ≤ 0.05.

**Table 3:** Hormonal assessment before and after HM evacuation.

	Cases A (*n* = 23)	Cases B (*n* = 23)	Cases C (*n* = 23)	*P* = A versus B	*P* = A versus C
	(0 w)	(48 hrs)	(2 w)		
FSH (UI/L)	0.1 ± 0.02	0.14 ± 0.12	2.79 ± 2.33	0.88	<0.001^*^
LH (IU/L)	0.1 ± 0 .03	0.11 ± 0.05	4.82 ± 2.85	0.60	<0.001^*^
hCG (mIU/mL)	126,897 ± 39,761	26,636 ± 18,155	209.2 ± 104.9	<0.001^*^	<0.001^*^
TST (ng/dL)	126.19 ± 57.35	105.21 ± 49.89	52.50 ± 32.90	0.18	<0.001^*^
FTST (pg/mL)	2.78 ± 1.24	1.54 ± 0.71	1.25 ± 0.77	0.002^*^	<0.001^*^
AN (ng/mL)	3.88 ± 2.22	3.02 ± 1.67	2.93 ± 1.98	0.11	0.04^*^
DHEA-S (*μ*/dL)	231.48 ± 124.59	161.43 ± 71.68	137.39 ± 55.96	0.009^*^	0.005^*^
Cortisol (nmol/L)	181.70 ± 111.08	121.75 ± 56.40	174.09 ± 70.15	0.04^*^	0.78
TSH (mU/L)	0.81 ± 0.69	0.67 ± 1.03	1.86 ± 1.01	0.86	<0.001^*^
T4 (*μ*g/dL)	13.98 ± 4.97	12.19 ± 3.65	9.87 ± 2.02	0.02^*^	0.001^*^
FT4 (ng/dL)	1.73 ± 1.12	1.46 ± 0.55	1.06 ± 0.17	0.06	0.01^*^
T3 (ng/dL)	228.88 ± 93.87	135.90 ± 38.69	157.58 ± 29.01	0.001^*^	0.01^*^

HM: hydatidiform mole; W: week; FSH: follicle-stimulating hormone; LH: luteinizing hormone; hCG: human chorionic gonadotropin; TTST: total testosterone; FTST: free testosterone; AN: androstenedione; DHEA-S: dehydroepiandrosterone sulfate; TSH: thyroid stimulating hormone; T4: thyroxin; FT4: free thyroxin; T3: liothyronine.

Plus-minus values are mean ± standard deviation.

^*^Significant P ≤ 0.05.

**Table 4:** Cases versus controls 2 weeks after HM evacuation.

	Cases (16 w)	Controls	*P* value
TST (ng/dL)	52.50 ± 32.90	104.02 ± 33.27	0.001^*^
FTST (pg/mL)	1.25 ± 0.77	1.50 ± 0.75	0.25
AN (ng/mL)	2.93 ± 1.98	3.55 ± 1.42	0.23
DHEA-S (*μ*/dL)	137.39 ± 55.96	133.59 ± 60.69	0.82
Cortisol	174.09 ± 70.15	188.53 ± 97.72	0.56
TSH (mU/L)	1.86 ± 1.01	2.69 ± 3.71	0.30
T4 (*μ*g/dL)	9.87 ± 2.02	11.84 ± 1.91	0.001^*^
FT4 (ng/dL)	1.06 ± 0.17	1.07 ± 0.14	0.88
T3 (ng/dL)	157.58 ± 29.01.12	181.60 ± 34.04	0.01^*^

HM: hydatidiform mole; hCG: human chorionic gonadotropin; TTST: total testosterone; FTST: free testosterone; AN: androstenedione; DHEA-S: dehydroepiandrosterone sulfate; TSH: thyroid stimulating hormone; T4: thyroxin; FT4: free thyroxin; T3: liothyronine.

Plus-minus values are mean ± standard deviation.

^*^Significant *P* ≤ 0.05.

## Abbreviations

GTD: Gestational trophoblastic disease
HM: Hydatidiform mole
TSH: Thyroid stimulating hormone
FSH: Follicle-stimulating hormone
LH: Luteinizing hormone
T: Testosterone
DHEA-S: Dehydroepiandrosterone sulfate
BMI: Body mass index
T4: Total thyroxin
FT4: Free thyroxin
T3: Liothyronine.
